# Long working hours and pregnancy complications: women physicians survey in Japan

**DOI:** 10.1186/1471-2393-14-245

**Published:** 2014-07-23

**Authors:** Masumi Takeuchi, Mahbubur Rahman, Aya Ishiguro, Kyoko Nomura

**Affiliations:** Teikyo University Support Center for Women Physicians and Researchers, 2-11-1 Kaga, Itabashi-ku, 173-8605 Tokyo, Japan; Center for Interdisciplinary Research in Women’s Health, Department of Obstetrics and Gynecology, University of Texas Medical Branch, 301 University Blvd, Galveston, Texas 77555 USA; Department of Hygiene and Public Health, Teikyo University School of Medicine, 2-11-1 Kaga, Itabashi-ku, Tokyo, 173-8605 Japan

**Keywords:** Pregnancy complication, Preterm birth, Threatened abortion, Women physicians, Working hours

## Abstract

**Background:**

Previous studies have investigated the impact of occupational risk factors on health outcomes among physicians. However, few studies have investigated the effects on pregnancy outcomes among physicians. In this study, we examined the association between working hours during pregnancy and pregnancy complications among physicians.

**Methods:**

A cross-sectional study was based on a survey conducted in 2009-2011 of 1,684 alumnae (mean age, 44 ± 8 years) who had graduated from 13 private medical schools in Japan. Data on threatened abortion (TA), preterm birth (PTB), and the number of working hours during the first trimester of pregnancy were obtained via retrospective assessments.

**Results:**

Of the 939 physicians with a first pregnancy, 15% experienced TA and 12% experienced PTB. Women who experienced TA (mean weekly working hours: 62 h vs. 50 h, *P* < .0001) or PTB (62 h vs. 50 h, *P* < .0001) had longer weekly working hours during the first trimester than did those without pregnancy complications. Compared with women who worked 40 hours or less per week, women who worked 71 hours or more per week had a three-fold higher risk of experiencing TA (95% confidence interval (CI): 1.7-6.0) even after adjusting for medical specialty, maternal age, and current household income. The risk of experiencing PTB was 2.5 times higher (95% CI:1.2-5.2) in women who worked 51-70 hours and 4.2 times higher (95% CI: 1.9-9.2) in women who worked 71 hours or more even after adjusting for specialty, maternal age, and current household income. The trend in the P statistic reflecting the effect of the quartile of hours worked per week (40 hours, 41-50 hours, 51-70 hours, ≥71 hours) on TA or PTB was 0.0001 in the multivariate logistic regression models.

**Conclusion:**

These results suggest that working long hours during the first trimester of pregnancy is associated with TA and PTB.

**Electronic supplementary material:**

The online version of this article (doi:10.1186/1471-2393-14-245) contains supplementary material, which is available to authorized users.

## Background

Physicians are susceptible to numerous unhealthy risks due to myriad occupational risk factors, such as anesthetic gases, toxic chemicals, prolonged standing, lifting heavy items, psychological stress, long working hours, and frequent on-call duty. Previous studies have demonstrated that sleep deprivation due to long working hours increases medical error rates among residents [1], and this finding was part of the impetus to enact ≤80-h/week work limits in the US [2]. Even after these regulations were enacted, studies have showed that extended work shifts were associated with increased risk of automobile accidents [3] and percutaneous needle-stick injuries [4] among residents. Indeed many countries recommend maximum working hours: 40 h in Japan (Article 66 of the Labor Act) and in the US (California Labor Laws), and 48 h in the UK (Working Time Regulations) and EU countries (Directive 2003/88/EC of the European Parliament and of the Council of 4 November 2003).

Although several studies [5–10] have investigated the potential association between working hours and pregnancy complications among working women, many of these studies did not take into account an occupation except for a study among physicians [11]. The demographic characteristics of the physician workforce have changed dramatically over the past three decades. The number of women entering medicine has increased in the past several decades, reaching nearly 50% of the total medical school enrollment in the US [12] and nearly 40% of the total number of physicians in Western countries [13]. Thus, pregnancy during residency or fellowship is not uncommon among physicians. This demographic shift toward more women in medicine warrants research on the risks encountered by pregnant physicians, as very few recent studies have examined this important topic [11]. Hence, we aimed to investigate the association between number of hours worked and pregnancy complications in physicians.

## Methods

### Subjects

**Figure 1:**
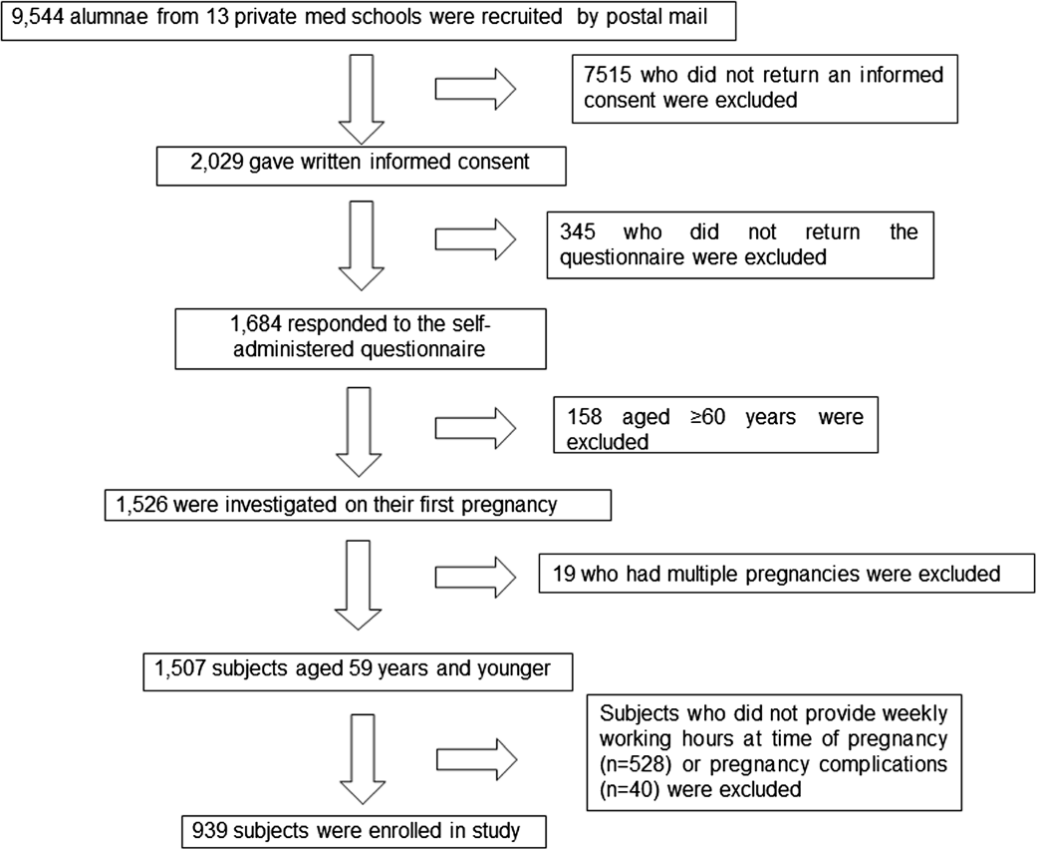
**Flowchart of the study population.**

This cross-sectional study was conducted between June 2009 and May 2011 using a self-administered survey completed by alumnae who graduated from 13 private medical schools in Japan. Japan has 80 medical schools, 29 of which are private. The present study was conducted in collaboration with the Council of Private Medical School Alumni Associations. We aimed to identify the barriers encountered by women as they tried to balance work and expected gender roles, because previous research has shown that physicians tend to switch from full-time to part-time at the time of life events of pregnancy and child-rearing which leads to career stagnation [5]. Of the 18 schools in the Eastern region, 13 agreed to participate in the study. In total, we contacted 9,544 alumnae from 13 medical schools via mail. Of these, 2,029 provided written informed consent, and 1,684 alumnae returned their questionnaires (Figure 1). A total of 745 physicians were excluded because they were aged ≥60 years (*n* = 158), or because data on working hours during their first pregnancy (*n* = 528) or pregnancy complications (*n* = 40) were not available. Additionally, because women with multiple conceptions (a situation in which two or more fetuses are conceived at the same time in the same woman) are more likely to experience adverse pregnancy complications, including preterm birth (PTB) or bleeding [14], the majority of previous studies restricted their sample to women with singleton pregnancies. Thus, we also excluded women with multiple conceptions (*n* = 19). Accordingly, 939 physicians (mean age: 44 ± 8 years) were included in the final analysis.

All participants provided written informed consent and were blind to the research hypotheses. All procedures were approved by the Institutional Review Board of Teikyo University School of Medicine (No. 08-107).

### Questionnaire

The details of the questionnaire are described elsewhere [15]. The items investigated in this study were: age at time of survey, medical specialty, maternal age at the time of first pregnancy, current household income at time of survey, weekly working hours during the first trimester of the first pregnancy, and complications during the first pregnancy. The number of hours worked per week during the first trimester was obtained by asking “On average, how many hours per week did you work when you initially became aware of your first pregnancy?”. Self-reported pregnancy complications during the first pregnancy were categorized into threatened abortion (TA), PTB, abnormal labor and delivery, spontaneous miscarriage, gestational hypertension, and others. TA was defined as vaginal bleeding in the first 22 weeks accompanied by a medical diagnosis of TA. PTB was defined as the delivery of a baby before 37 weeks of gestation. Abnormal labor and delivery included anomalies in rotation, hemorrhaging due to abruption placentae, looping or coiling of the umbilical cord, atonic bleeding, cephalopelvic disproportion, cervical incompetence, placenta previa, and low-lying placenta. Gestational hypertension or pregnancy-induced hypertension was defined as the development of new arterial hypertension in a pregnant woman after 20 weeks gestation, without the presence of protein in the urine. Other complications included underlying gynecological illnesses, such as uterine myoma and endometriosis, severe nausea, and other complications involving internal organs (e.g., diabetes and tuberculosis). Among these categories, TA and PTB were considered as primary outcomes of interest in this study. A recent review in this field suggested that PTB may be associated with long working hours during pregnancy [5], and a recent meta-analysis based on 14 studies indicated that first-trimester bleeding was associated with significantly higher rates of perinatal mortality and low-birth weight babies [16]. Current household income was measured by asking, “In which class does your household income place you?” Responses were classified into quintiles from the highest to the lowest (Additional file 1).

### Statistical analysis

Analyses of weekly working hours during the first trimester of pregnancy by pregnancy complications were performed using Student’s t-test. Associations between the quartile of number of hours worked per week and pregnancy complications were investigated using *chi*-square tests. Unadjusted and age-adjusted odds ratios (ORs) of the effect of quartile of hours worked per week on TA and PTB were calculated with logistic regression models, and 95% confidence intervals (CIs) were determined. We adjusted for quartile of maternal age, medical specialty, and quintile of current household income in the multivariate analyses. We adjusted for medical specialty because previous studies have suggested that occupational exposure to anesthetics may be dangerous to the health of the fetus and mother [17, 18] and that night on-call duty or prolonged standing, which are typical among surgeons may increase pregnancy complications [5]. In addition, we adjusted for current household income because a previous study in the US found that an association between an extended work week and higher monetary income, which indicates that women in higher socioeconomic status are more likely to work longer. Medical specialty was further categorized into three groups: "surgery", which included general surgery, otorhinolaryngology, dermatology, urology, OBGYN, opthalmology, orthopedic surgery, plastic surgery, cosmetic surgery, and neurosurgery; "general medicine", which included internal medicine, pediatrics, psychosomatic medicine, family medicine, neurology, and palliative care; and “other", which included psychiatry, anesthesiology, radiology, basic medicine, rehabilitation, emergency medicine, and those who had not yet chosen a specialty yet. All analyses were conducted using SAS software (version 9.12; Cary, NC), and statistical significance was set at p <0.05. Analyses of all pregnancy complications examined any pregnancy complication (i.e., they are not mutually exclusive); those focused on only TA (or PTB) excluded subjects with PTB (or TA).

## Results

Table 1 indicates subject characteristics. The mean age of the sample was 44 ± 8 years and the mean maternal age during the first pregnancy was 31 ± 4 years. The mean number of hours worked per week during the first trimester of the first pregnancy was 54 ± 22 hours. Of the 939 subjects, 394 (42%) reported at least one complication during their first pregnancy. TA (*n* = 93, 15%) and PTB (*n* = 71, 12%) were the most frequent complications, and 36 participants experienced both TA and PTB.

**Table 1:** **Subject characteristics (n = 939)**

			N	%
Age at survey, years				
	≤39		314	33
	40-48		306	33
	49≤		319	34
Maternal age, years				
	<28		234	25
	28-29		181	19
	30-32		259	28
	33≤		265	28
Specialty				
Internal medicine			283	30
	Dermatology		111	12
	Pediatrics		104	11
	Opthalmology		102	11
	OBGY		65	7
	Psychiatry		51	5
	Ear nose Throat		50	5
	Anesthesiology		35	4
	Has not decided yet		26	3
	Others^a^		110	12
Any pregnancy complications				
	Overall^b^		394	42
		Threatened abortion	129	14
		Preterm birth	107	11
		Abnormal labor and delivery	72	8
		Spontaneous miscarriage	26	3
		Gestational hypertension	26	3
		Others^c^	98	10
	No complication		541	58
	Do not remember		4	0
Current household income				
	Highest quintile		142	18
	Second highest quintile		417	52
	Middle		137	17
	Second lowest quintile		71	9
	Lowest quintile		30	4
Weekly working hours during the first trimester, mean (SD)				54 (22) hrs

SD (standard deviation); The numbers in each category may not add up to the total if the data contain missing values.

^a^Others include Rehabilitation, Psychosomatic medicine, Palliative Care, Neurosurgery, Emergency medicine, Urology, Cosmetic surgery, Cardiovascular surgery, Surgery, Neurology, Orthopedic surgery, Family medicine, Plastic surgery, Radiology, Basic medicine.

^b^Pregnancy complications were not mutually exclusive here.

^c^Others included gynecological underlying illnesses such as uterine myoma and endometriosis, severe nausea, and other complications with internal organs like diabetes and tuberculosis.

Table 2 shows the number of hours worked per week during the first pregnancy. Women who experienced TA (mean: 63 h *vs.* 50 h, *P* <0.0001) or PTB (mean: 62 h *vs.* 50 h, *P* <0.0001) had longer working weeks during pregnancy than did women without complications. However, the mean number of hours worked by women who had complications other than TA or PTB did not differ from the hours worked by those who had no complications at all.

**Table 2:** **Weekly working hours during the first trimester of pregnancy by any pregnancy complications**

Variables			Weekly working hours at pregnancy	P ^a^
			Mean (SD)	
Any pregnancy complications				
	Overall		58 (23) hrs	<.0001
		Threatened abortion	63 (22) hrs	<.0001
		Preterm birth	62 (25) hrs	0.0003
	No complication		50 (21) hrs	Reference^b^

^a^Based on t test.

^b^Those who answered "do not remember" were excluded (n = 4).

**Figure 2:**
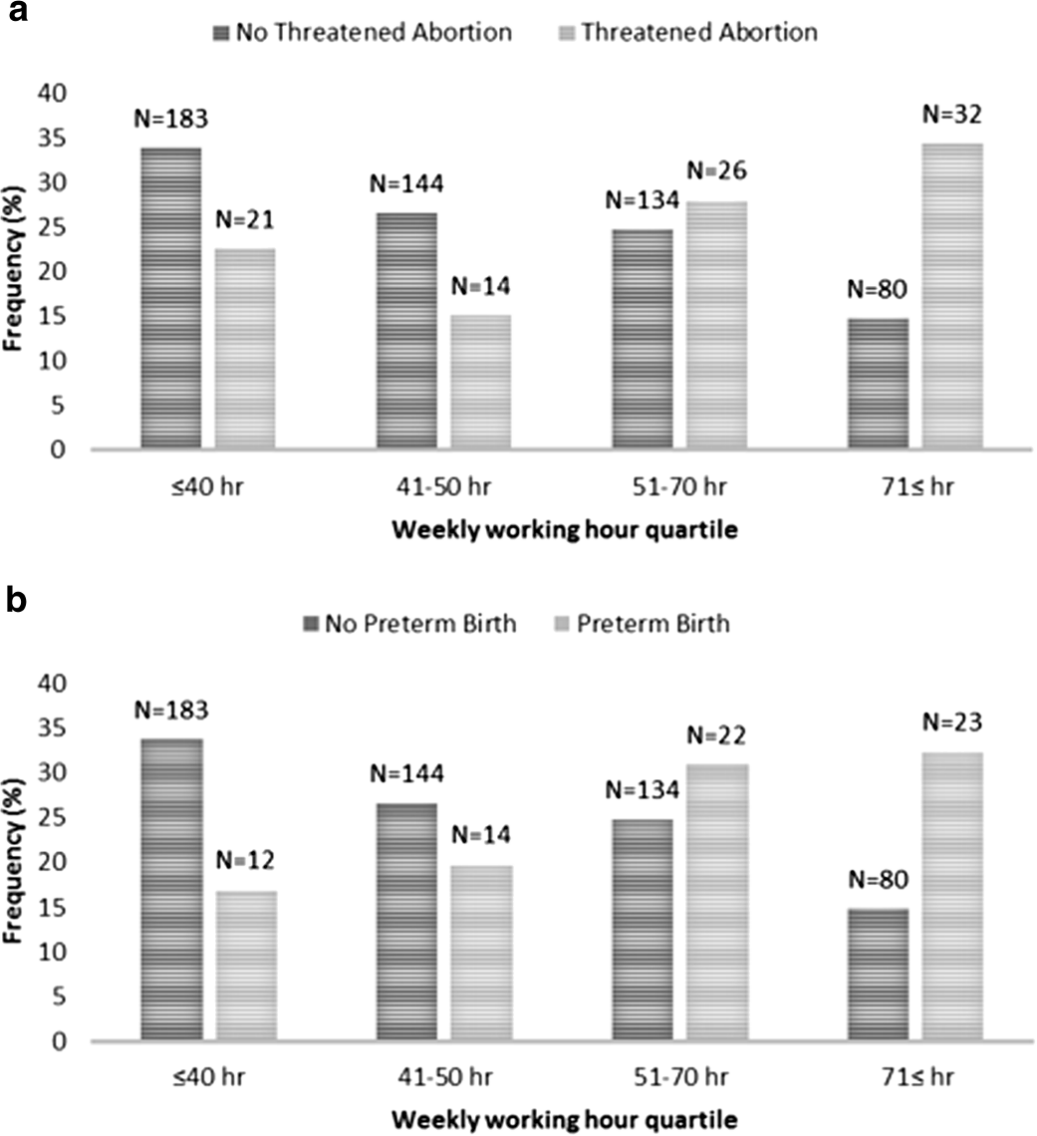
**Frequency of threatened abortion (TA) and no threatened abortion, preterm birth (PTB) and no preterm birth, according to weekly working hour quartile.** The frequency of TA and PTB increased as the number of working hours increased (*P* based on *chi*-square test <0.0001 for both trends).

Figure 2 shows the frequency of TA (Figure 2a) or PTB (Figure 2b) for each quartile of hours worked per week during the first pregnancy. A total of 275 women (29.3%) worked ≤ 40 hours/week, 232 (24.7%) worked 41-50 hours/week, 247 (26.3%) worked 51-70 hours/week, and 185 (19.7%) worked ≥71 hours/week. The number of women experiencing TA or PTB increased as each quartile of hours worked per week increased from the lowest to the highest (*P* based on *chi*-square test <0.0001 for both trends).

The odds ratios for TA by logistic regression models were presented in Table 3. Compared with women who worked 40 hours or less per week, women who worked 71 hours or more per week had a three-fold higher risk of TA (95% CI: 1.7-6.0) even after adjusting for specialty, maternal age, and current household income. The risk of experiencing TA was 0.9 (95% CI: 0.4-1.8) for those working 41-50 hours/week and 1.7 (95% CI: 0.9-3.1) for those working 51-70 hours/week. Although these point estimates increased in a linear fashion (trend *p* = 0.0001), they were not statistically significant.

**Table 3:** **Odds ratios of weekly working hours on threatened abortion**

		Crude		Adjusted	
		OR	95% CI	OR	95% CI
Weekly working hours					
	71≤	3.49	(1.89-6.42)	3.17	(1.69-5.95)
	51-70	1.69	(0.91-3.13)	1.67	(0.89-3.12)
	41-50	0.85	(0.42-1.72)	0.88	(0.43-1.80)
	≤40	1.00	―	1.00	―
Specialty group^a^					
	Surgery	0.64	(0.34-1.23)	0.63	(0.32-1.23)
	General medicine	1.04	(0.56-1.91)	0.88	(0.47-1.66)
	Others	1.00	―	1.00	―
Maternal age, years					
	33≤	0.55	(0.31-1.00)	0.77	(0.39-1.53)
	30-32	0.55	(0.31-1.00)	0.73	(0.37-1.45)
	28-29	0.73	(0.38-1.38)	1.02	(0.49-2.10)
	<28	1.00	―	1.00	―
Current household income					
	Lowest quintile	2.19	(0.69-6.95)	1.50	(0.43-5.22)
	Second lowest quintile	2.78	(1.22-6.33)	2.19	(0.90-5.31)
	Middle	1.49	(0.66-3.35)	1.48	(0.64-3.40)
	Second highest quintile	1.33	(0.67-2.61)	1.34	(0.67-2.68)
	Highest quintile	1.00	―	1.00	―

"Crude" incdicates univariate analyses and "Adjusted" indicates all variables in the model; Subjects with preterm birth were excluded in the model where threatened abortion was dependent variable; Similarly, subjects with threatened abortion were excluded in the model where preterm birth was dependent variable.

^a^Specialty group consists of "Surgery" including General Surgery, Otorhinolaryngology, Dermatology, Urology, OBGY, Opthalmology, Orthopedic Surgery, Plastic Surgery, Cosmetic Surgery, and Neurosurgery, "General Medicine" including Internal medicine, Pediatrics, Psychosomatic Medicine, Family Medicine, Neurology, and Palliative Care, and “Others” including Psychiatry, Anesthesiology, Radiology, Basic medicine, Rehabilitation, Emergency Medicine, and those who has not chosen specialty yet.

The odds ratios for PTB by logistic regression models are presented in Table 4. Compared with women who worked 40 hours a week or less, the risk of experiencing PTB was 2.5-fold higher (95% CI: 1.2-5.2) among women who worked 51-70 hours and 4.2-fold higher (95% CI: 1.9-9.2) among women who worked 71 hours or more even after adjusting for specialty, maternal age, and current household income. Although the odds ratio of PTB for women who worked 41-50 hours was 1.44 (95% CI: 0.63-3.31), which was not statistically significant, the trend in the *P*-value for the effect of quartile of weekly working hours on PTB was 0.0001 in the multivariate logistic regression models.

**Table 4:** **Odds ratios of weekly working hours on preterm birth**

		Crude		Adjusted	
		OR	95% CI	OR	95% CI
Weekly working hours					
	71≤	4.38	(2.08-9.24)	4.19	(1.91-9.21)
	51-70	2.50	(1.20-5.24)	2.46	(1.16-5.23)
	41-50	1.48	(0.67-3.30)	1.44	(0.63-3.31)
	≤40	1.00	―	1.00	―
Specialty group^a^					
	Surgery	0.93	(0.44-1.95)	0.74	(0.34-1.62)
	General medicine	1.11	(0.54-2.31)	0.86	(0.40-1.83)
	Others	1.00	―	1.00	―
Maternal age, years					
	33≤	0.78	(0.37-1.66)	1.23	(0.51-3.00)
	30-32	1.14	(0.57-2.30)	1.65	(0.71-3.83)
	28-29	1.59	(0.77-3.28)	2.39	(0.99-5.75)
	<28	1.00	―	1.00	―
Current household income					
	Lowest quintile	2.92	(0.88-9.69)	2.90	(0.76-11.08)
	Second lowest quintile	0.93	(0.27-3.16)	0.96	(0.27-3.44)
	Middle	2.51	(1.08-5.85)	2.38	(1.00-5.66)
	Second highest quintile	1.22	(0.56-2.65)	1.06	(0.48-2.35)
	Highest quintile	1.00	―	1.00	―

"Crude" incdicates univariate analyses and "Adjusted" indicates all variables in the model; Subjects with preterm birth were excluded in the model where threatened abortion was dependent variable; Similarly, subjects with threatened abortion were excluded in the model where preterm birth was dependent variable.

^a^Specialty group consists of "Surgery" including General Surgery, Otorhinolaryngology, Dermatology, Urology, OBGY, Opthalmology, Orthopedic Surgery, Plastic Surgery, Cosmetic Surgery, and Neurosurgery, "General Medicine" including Internal medicine, Pediatrics, Psychosomatic Medicine, Family Medicine, Neurology, and Palliative Care, and “Others” including Psychiatry, Anesthesiology, Radiology, Basic medicine, Rehabilitation, Emergency Medicine, and those who has not chosen specialty yet.

## Discussion

This study demonstrated that the number of hours worked per week during the first trimester of pregnancy was associated with pregnancy complications such as TA (partially) and PTB. Compared with women who worked 40 hours or less per week, women who worked 71 hours or more per week had a three-fold higher risk of experiencing TA, women who worked 51-70 hours per week had 2.5 times higher risk of experiencing PTB, and women who worked 71 hours or more had 4.2 times higher risk of experiencing PTB even after adjusting for medical specialty, maternal age, and current household income.

The results of related published studies have not been consistent. For example, Pompeii et al. [6] observed a protective effect of working hours on PTB. Several studies [7–11, 17] reported that long working hours increased the risk of PTB, whereas others failed to identify any association in this regard [5]. Inconsistent results across studies may be primarily attributable to the small effect size of the number of working hours on PTB, different cut-off points for working hours per week, data collection at different time points during pregnancy (e.g., different trimesters), or the confounding effects of occupation and different job characteristics. In terms of the effect of occupation, there was only one study that focused on women physicians, published in 1990s. This study found that residents who worked ≥100 hours per week during the first trimester of pregnancy had a higher risk of preterm delivery compared with residents who worked < 100 hours per week (9.8% vs. 4.6%, *P* = 0.012). Taken together, this previous study and our research adds to the literature showing that longer working hours may increase the risk of preterm delivery among physicians.

The prevalence of TA and PTB in our sample was 15% and 12%, respectively, which were close to the rates in the general population (i.e., those of TA and PTB have been reported as 16-25% [19] and 11% [20], respectively). It has previously been recognized that maternal age is associated with adverse pregnancy complications. In our sample, the mean maternal age during the first pregnancy was 31 ± 4 years, which is slightly older than that in Japanese general population (i.e., mean = 29.7 years based on the 2010 Vital statistics reported by the Japanese Ministry of Health, Labour, and Welfare). Thus, the comparable prevalence found by the present study and previous reports [19, 20] may be explained by the small difference between the maternal age of between our sample and general population. Alternatively, our sample may have been healthier than the general population.

This study also has several limitations. First, our sampling methods may have resulted in sampling bias and selection bias. Sampling bias may have arisen from our inclusion of only private medical school alumnae, and from the low participation rate (1,684 of the 9,544 subjects who were initially recruited for this study actually participated). This sampling bias may undermine the external validity of our results, and our findings may not be generalizable to all women physicians in Japan. Selection bias may have been caused by a tendency of participants who were frustrated with poor working conditions to over-report the number of hours worked per week. Because such a selection bias would undermine internal validity, our results should be interpreted cautiously. Second, we measured the number of hours worked per week during the first trimester by asking “On average, how many hours per week did you work when you initially became aware of your first pregnancy?” Although, menstruation stops after a woman gets pregnant, some women continue to experience some hormone-driven or abnormal bleeding while pregnant, and mistakenly perceive it as menstruation. Thus, some women may have answered this question with reference to their second trimester, which may have caused misclassification. Third, self-reported data may be subject to recall bias. Physicians may be aware that long working hours may be detrimental to pregnancy, and they may have unconsciously added hours to their first trimester work experience after experiencing pregnancy complications. To examine the extent of recall bias, we performed sensitivity analyses by excluding women who were ≥45 years of age from the analyses, but the results did not change. According to the additional analysis, subjects with TA or PTB were more likely to work longer hours (i.e., 60 or 65 h per week, respectively) compared with those without complications (i.e., 50 h per week). Additionally, the self-reported rates of TA and PTB were similar to those in previous studies, suggesting that recall bias may not be critical. Fourth, although the outcome variables (i.e., TA and PTB) in this study were defined based on medical diagnosis, our use of a self-report measure may have resulted in the over-reporting of unspecified symptoms, such as vaginal bleeding, as TA. Thus, the frequency of TA may have been overestimated. Fifth, we collected data regarding the first trimester of pregnancy, which is when the rates of fetal growth and development are considered to be highest [21]. However, previous research has suggested that the last trimester is more strongly associated with PTB [22]. Future studies should investigate the impact of number of hours worked on pregnancy outcome according to trimester. Sixth, we did not measure lifestyle variables, such as alcohol intake, caffeine consumption, or smoking. However, these factors have been reported to cause short fetal crown-to-rump length, which has been associated with babies who are small for their gestational age [23], but not with PTB. However, we still did not adjust for a lifestyle factor of physical activity or even vaginal infection which may contribute to preterm birth but was not included in the analysis. Seventh, the demands placed upon physicians can be detrimental and possibly dangerous to the health of the fetus and the mother. For example, a previous report [18] highlighted the reproductive risks related to occupational exposure to anesthetics. Therefore, one analysis, excluded women whose specialty included anesthesiology but the results did not change. Finally, unmeasured factors, such as stress, fatigue, or the psychological burden related to long working hours, may have affected our results.

## Conclusions

Despite these limitations, the results of this study demonstrate that long working hours during the first trimester are associated with pregnancy complications among physicians. Currently, very few professional guidelines include limits on the number of hours worked. In 2003, the American Accreditation Council for Graduate Medical Education recommended that residents be limited to 80 hours of work per week [2]. However, it did not specify working hours for pregnant physicians. Pregnancy during residency is common given that lengthy medical training overlaps with the main childbearing years. Therefore, future research using a cohort study design could contribute to legal or professional regulations governing the number of hours pregnant physicians can work by investigating whether long working hours cause TA or PTB.

## Authors’ information

MT is a researcher of Teikyo University Support Center for Women Physicians and Researchers, RM is an associate professor of Center for Interdisciplinary Research in Women’s Health, Department of Obstetrics and Gynecology, University of Texas Medical Branch. AI is an instructor of Department of Hygiene and Public Health, Teikyo University School of Medicine. KN is a director of Teikyo University Support Center for Women Physicians and Researchers, and an associate professor of Department of Hygiene and Public Health, Teikyo University School of Medicine.

## Electronic supplementary material

Additional file 1: **Appendix.** Item used in *Questionnaire.* (DOCX 18 KB)

## Abbreviations

TA: Threatened abortion
PTB: Preterm birth
SD: Standard deviation
OR: Odds ratio
CI: Confidence intervals.
