# Supplementary material for: Long working hours and pregnancy complications: women physicians survey in Japan
Source: BMC Pregnancy Childbirth. 2014 Jul 23;14:245. doi: 10.1186/1471-2393-14-245 (PMC4121483; doi:10.1186/1471-2393-14-245)
Supplement: Supplementary file 1 — Additional file 1: Appendix. Item used in Questionnaire. (DOCX 18 KB) [file 12884_2014_1114_MOESM1_ESM.docx]

Appendix. Item used in *Questionnaire*

Item 1 Age at survey

Item 2 Maternal age at the time of first pregnancy that was computed by subtracting age of first child from maternal age at survey

Item 3 Medical specialty

Item 4 Current household income at time of survey,

“In which class does your household income place you?”


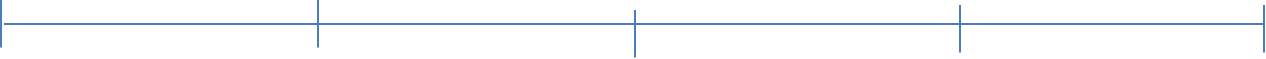


1 2 3 4 5

Lowest fifth Second fifth Middle fifth Fourth fifth Highest fifth

Item 5 The number of hours worked per week during the first trimester

“On average, how many hours per week did you work when you initially became aware of your first pregnancy?”

Item 6 Pregnancy complications

“Did you have any abnormalities or complications during pregnancy or labor and birth? If you answered “yes”, what was it based on medical diagnosis (please describe it)?”
